# Bioinformatics Analysis of a Prognostic miRNA Signature and Potential Key Genes in Pancreatic Cancer

**DOI:** 10.3389/fonc.2021.641289

**Published:** 2021-05-20

**Authors:** Shuoling Chen, Chang Gao, Tianyang Yu, Yueyang Qu, Gary Guishan Xiao, Zunnan Huang

**Affiliations:** ^1^ Key Laboratory of Big Data Mining and Precision Drug Design of Guangdong Medical University, Key Laboratory for Research and Development of Natural Drugs of Guangdong Province, School of Pharmacy, Guangdong Medical University, Dongguan, China; ^2^ The Second School of Clinical Medicine, Guangdong Medical University, Dongguan, China; ^3^ Southern Marine Science and Engineering Guangdong Laboratory (Zhanjiang), Zhanjiang, China; ^4^ School of Pharmaceutical Science and Technology, Dalian University of Technology, Dalian, China

**Keywords:** pancreatic cancer, miRNAs, biomarkers, target genes, The Cancer Genome Atlas, Gene Expression Omnibus

## Abstract

**Background:**

In this study, miRNAs and their critical target genes related to the prognosis of pancreatic cancer were screened based on bioinformatics analysis to provide targets for the prognosis and treatment of pancreatic cancer.

**Methods:**

R software was used to screen differentially expressed miRNAs (DEMs) and genes (DEGs) downloaded from The Cancer Genome Atlas (TCGA) and Gene Expression Omnibus (GEO) databases, respectively. A miRNA Cox proportional hazards regression model was constructed based on the miRNAs, and a miRNA prognostic model was generated. The target genes of the prognostic miRNAs were predicted using TargetScan and miRDB and then intersected with the DEGs to obtain common genes. The functions of the common genes were subjected to Kyoto Encyclopedia of Genes and Genomes (KEGG) and Gene Ontology (GO) analyses. A protein-protein interaction (PPI) network of the common genes was constructed with the STRING database and visualized with Cytoscape software. Key genes were also screened with the MCODE and cytoHubba plug-ins of Cytoscape. Finally, a prognostic model formed by the key gene was also established to help evaluate the reliability of this screening process.

**Results:**

A prognostic model containing four downregulated miRNAs (hsa-mir-424, hsa-mir-3613, hsa-mir-4772 and hsa-mir-126) related to the prognosis of pancreatic cancer was constructed. A total of 118 common genes were enriched in two KEGG pathways and 33 GO functional annotations, including extracellular matrix (ECM)-receptor interaction and cell adhesion. Nine key genes related to pancreatic cancer were also obtained: MMP14, ITGA2, THBS2, COL1A1, COL3A1, COL11A1, COL6A3, COL12A1 and COL5A2. The prognostic model formed by nine key genes also possessed good prognostic ability.

**Conclusions:**

The prognostic model consisting of four miRNAs can reliably predict the prognosis of patients with pancreatic cancer. In addition, the screened nine key genes, which can also form a reliable prognostic model, are significantly related to the occurrence and development of pancreatic cancer. Among them, one novel miRNA (hsa-mir-4772) and two novel genes (COL12A1 and COL5A2) associated with pancreatic cancer have great potential to be used as prognostic factors and therapeutic targets for this tumor.

## Background

Pancreatic cancer, also known as pancreatic ductal adenocarcinoma (PDAC), is a malignancy that frequently appears in the digestive system, and its incidence is on the rise worldwide (1). According to data published recently, PDAC has become the 10th most common malignant tumor, ranking 4th among the causes of death among malignant tumor patients (2). In China, PDAC is one of the major tumors whose both incidence and mortality are increasing (3).

The best and only radical treatment for PDAC is surgical resection (4). However, for many years, there has been no significant improvement in the surgical resection rate or annual survival rate after surgical treatment (5). Moreover, due to extensive metastasis at the time of diagnosis, most patients miss the optimal time for surgery, and PDAC is not sensitive to radiotherapy and chemotherapy. The lack of proper treatment methods highlights the importance of the identification of new therapeutic targets for PDAC. As the study of miRNAs has deepened in recent years, an increasing number of miRNAs have been confirmed to be related to the development of cancers, including PDAC (6, 7). Therefore, it is of great importance to further clarify how miRNAs affect the pathogenesis, invasion and metastasis of PDAC and to provide novel treatment methods.

In recent years, it has been reported that microRNAs (miRNAs, miRs) are influencing factors of PDAC. For example, He et al. (8) illustrated that overexpressed miR-371-5p is associated with a poor prognosis in PDAC patients, and miR-371-5p inhibitors suppress the proliferation of PDAC cells by blocking the cell cycle (8). Deng et al. (9) demonstrated that the downregulation of miR-26a in PDAC cells can inhibit cyclin E2 expression, decreasing the patient survival rate. Zhao et al. (10) found that increasing the expression of miR-148b can suppress the expression of its target gene AMP-activated protein kinase α1 (AMPKα1) to inhibit metastasis and invasion while improving the chemosensitivity of PDAC cells. Therefore, miRNAs can be used as potential biomarkers for PDAC, and the range of their application is broad.

However, the process by which novel miRNA biomarkers are experimentally identified is time consuming and laborious, and the results are not necessarily ideal. Therefore, bioinformatics methods have been proposed to mine such markers from clinical data stored on the internet. The Cancer Genome Atlas (TCGA, https://portal.gdc.cancer.gov/) (11), Gene Expression Omnibus (GEO, http://www.ncbi.nlm.nih.gov/geo) (12), Database for Annotation, Visualization and Integrated Discovery (DAVID, https://david.ncifcrf.gov/) (13), STRING (http://string-db.org/cgi/input.pl) (14), R software (https://www.r-project.org/) (15) and Cytoscape (https://cytoscape.org/) (16) are popular databases and tools that can be used for data download, functional enrichment and protein-protein interaction (PPI) analysis. In recent years, studies have used bioinformatics methods to screen PDAC markers. Ye et al. (17) demonstrated that miR-7 showed predictive ability for PDAC, and lower miR-7 expression levels in patients lead to tumors with a more advanced stage as well as a worse prognosis. Borgmästars et al. (18) revealed that hsa-miR-885-5p acts as a tumor suppressor by calculation and predicted that it can act as a biomarker to predict the prognosis of PDAC patients.

In this study, by analyzing data from the TCGA and GEO databases using bioinformatics methods, we screened prognostic miRNAs and genes related to PDAC. R language packages and Cytoscape plug-ins were applied for the discovery of key genes that affect the occurrence of PDAC. The prognostic miRNAs and key genes we obtained may exert considerable impact on the progression of PDAC, which enables them to become potential therapeutic targets and to be considered for future investigations on PDAC. Our study may provide new ideas for future research on PDAC treatment.

## Materials and methods

### Data Download and Differential Expression Analysis

The miRNA transcriptome data with the clinical information of 183 pancreatic-related samples (179 tumor tissues and 4 normal tissues) were downloaded from the TCGA database (https://portal.gdc.cancer.gov/) on April 1, 2020. The GSE28735 dataset was directly downloaded from the GEO database (https://www.ncbi.nlm.nih.gov/geo/query/acc.cgi?acc=GSE28735) on April 1, 2020, and includes 45 tumor samples and 45 normal samples. In addition, its relevant clinical survival data were further retrieved from GEO2R website (https://www.ncbi.nlm.nih.gov/geo/geo2r/?acc=GSE28735) of GEO database on March 13, 2021. TCGA data were analyzed with the edgeR package, gplots package and limma package for DEMs with the screening criteria *P* < 0.05 and |log2FC| > 1.0. GEO data were analyzed by the limma package for DEGs with the same screening criteria.

### Construction of the Cox Proportional Hazards Regression Model

Univariate Cox proportional hazards regression analysis was performed on DEMs with survival package of R software. Then, with the criteria *P* < 0.05, DEMs from univariate Cox were selected and multivariate Cox stepwise regression analysis was performed on them with survival package of R software. After multivariate Cox, prognostic miRNAs were obtained, and prognostic miRNAs with *P* < 0.05 were considered independent prognostic factors.

### Establishment of a Prognostic Model

After the prognostic miRNAs were screened, a prognostic model based on the selected miRNAs was established, and we calculated the risk score of the model using the following formula: risk score = β1 × Exp (miRNA_1_) + β2 × Exp (miRNA_2_) +…+ βn × Exp (miRNA_n_). Subsequently, on the basis of the median risk score, patients were assigned to two different groups: high risk and low risk. Then, survival analysis was performed to establish a miRNA prognostic model. A risk score curve was plotted to demonstrate the risk score differences according to the model. A survival status map was plotted to demonstrate the survival status of every cancer sample. A heatmap was plotted to demonstrate the expression level of the prognostic miRNAs in every cancer sample and a survival curve was plotted to demonstrate the 3-year survival in the high- and low-risk groups. We also drew the ROC curve of the model. The AUC value of the model shows the predictive capability, and AUC value > 0.7 indicates the model has strong prognostic ability.

### Target Gene Prediction and Common Gene Acquisition

The online website databases TargetScan (http://www.targetscan.org/) (19) and miRDB (http://miRdb.org/) (20) were used to predict the target genes of the miRNAs from the prognostic model. To reduce the false positive rate, the target genes predicted by the two databases were intersected, and the overlapping genes from both databases were employed. Then, we intersected the target genes and the DEGs to obtain the common genes. At this time, the common genes were both the target genes of the prognostic miRNAs and the DEGs related to PDAC.

### Kyoto Encyclopedia of Genes and Genomes (KEGG) Pathway and Gene Ontology (GO) Functional Analyses of Common Genes

To further clarify the roles that the common genes play in biological processes, we used the DAVID (https://david.ncifcrf.gov/) to perform KEGG pathway enrichment and GO functional annotation analyses. The enriched KEGG pathway and GO functional annotations with *P* < 0.05 were obtained. The pathways and annotations with smallest *P* values and largest counts were considered crucial pathways and annotations. GO annotation includes three categories: biological process (BP), cellular component (CC) and molecular function (MF).

### Construction of the PPI Network and Screening of the Core Network

We used the online visualization tool STRING (http://string-db.org) to analyze interactions among the common genes. The PPI network was constructed with the common genes whose confidence score was greater than or equal to 0.400, and the disconnected genes were hidden. The network was then input into Cytoscape software (version 3.7.1, https://cytoscape.org/) for visualization. The logFC values of the genes in the network were also imported into Cytoscape. Key genes were screened using the MCC algorithm of the Cytoscape cytoHubba plug-in (21). Meanwhile, the functional modules of the common genes were scored and screened out using the Cytoscape MCODE plug-in with the following criteria: degree cut-off = 2, haircut on, node score cut-off = 0.2, k-core = 2, and max. depth = 100.

### MiRNA-Gene-Pathway Network Visualization

The targeted relationship network between the miRNAs and common genes and the pathways and annotations enriched in the common genes were also established using Cytoscape. The regulatory relationships among the miRNAs, key genes and enriched pathways of the common genes in the KEGG pathway analysis with the minimum *P* value or the maximum count value and the GO functional annotations are presented.

### Establish of a Prognostic Model Formed by Screened Key Genes

In order to evaluate the reliability of the key genes screened by Cytoscape, we directly established a prognostic gene model formed by those key genes. And the survival analysis of this new model was conducted, and the risk score curve, survival status map, heatmap and survival curve were plotted. The ROC curve was used as a criterion to show the predictive capability of this models and a AUC value > 0.7 also indicated a strong prognostic ability.

### Cell Culture

A normal human pancreatic ductal epithelial cell line was purchased from RiboBio Co., Ltd. (Guangzhou, China), and the PDAC cell lines SW1990 and PANC-1 were purchased from Shanghai GeneChem Co., Ltd. (Shanghai, China). SW1990 and PANC-1 cells were cultured in DMEM (Gibco Company, USA). HPDE6C7 cells were cultured in MEM medium (Gibco, USA). Both media contained 10% inactivated fetal bovine serum (Gibco, USA). All cells were incubated in an incubator at 37°C and 5% CO_2_.

### RNA Isolation and Real-Time Quantitative PCR

Total RNA was isolated from cultured cells using TRIzol reagent (Thermo Fisher Scientific, USA). A miRNA reverse transcription kit (RiboBio Co., Ltd, Guangzhou, China) was used to generate cDNA. A real-time quantitative PCR kit was used to conduct quantitative analysis. U6 was used as an endogenous control. The relative expression was analyzed by the 2^−ΔΔCt^ method. The primers used were as follows: hsa-mir-424: 5’-GCGCAGCAGCAATTCATGT-3’ and 5’-AGTGCAGGGTCCGAGGTATT-3’; and U6: 5’-CGCTTACGAATTTGCGTGTCAT-3’ and 5’-CTCGCTTCGGCAGCACA-3’.

## Results

### Differential Expression Analysis

**Figure 1:**
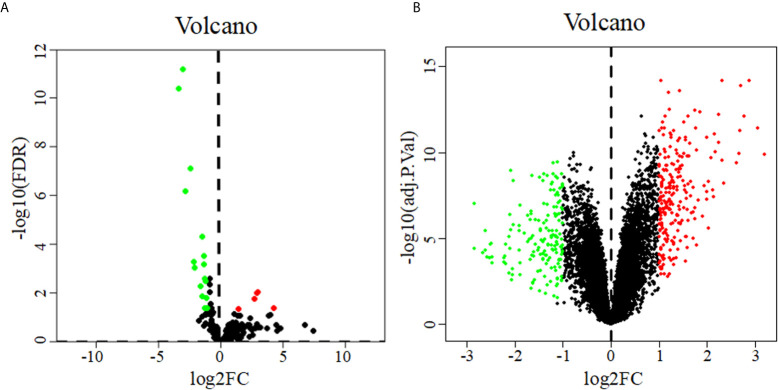
Volcano diagrams of DEMs **(A)** and DEGs **(B)**. Volcano diagrams show the P value and the fold change of differentially expressed miRNAs and genes. Green and red circles represent downregulated and upregulated miRNAs or genes, respectively.

22 DEMs were identified from 183 PDAC samples from the TCGA: 5 were upregulated, and 17 were downregulated (**Figure 1A**). A total of 402 DEGs were identified from the GSE28735 dataset of the GEO: 234 were upregulated, and 168 were downregulated (**Figure 1B**).

### Construction of the Cox Proportional Hazards Regression Model

**Figure 2:**
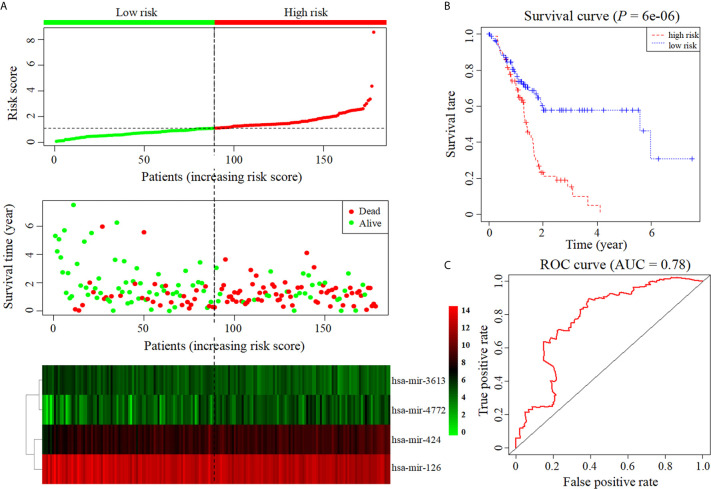
Construction of prognostic model based on four prognostic miRNAs. **(A)** The risk score curve, survival status and heatmap are shown from top to bottom. **(B)** Survival curve. **(C)**. ROC curve of the prognostic model.

Six miRNAs associated with the survival of PDAC patients were identified (*P* < 0.05) (**Table 1**). Four downregulated prognostic miRNAs were further selected (hsa-mir-424, hsa-mir-126, hsa-mir-3613 and hsa-mir-4772) (**Table 2**). Among them, the *P* values of hsa-mir-424, hsa-mir-126 and hsa-mir-3613 were less than 0.05, indicating that they were independent prognostic factors. The prognostic miRNA risk score was calculated according to the following formula: risk score = (0.6006 × hsa-mir-424) + (-0.6601 × hsa-mir-126) + (-0.3851 × hsa-mir-3613) + (0.1819 × hsa-mir-4772). Then, the samples were divided into a high-risk group and a low-risk group based on the medium risk score. The risk score of the former group was significantly higher than that of the latter group (**Figure 2A**, top). A high risk score correlated with a poor prognosis. Survival analysis showed that the mortality rate increased as the risk score increased (**Figure 2A**, middle). The heatmap showed that as the risk score increased, the expression levels of hsa-mir-4772, hsa-mir-424 and hsa-mir-126 increased, indicating that they were high-risk miRNAs; the expression of hsa-mir-3613 decreased as the risk score increased, indicating that it was a low-risk miRNA (**Figure 2A**, bottom). Compared with high-risk group, low-risk group survival rate was notably higher (P = 6e-06; three-year survival rate, low-risk group: 57.90%, 95% CI = 46.60%-71.90%; high-risk group: 15.21%; 95% CI = 7.69%-30.1%) (**Figure 2B**). The AUC value of the ROC curve of the model was 0.78 (**Figure 2C**), which was greater than 0.7, indicating that the model was reliable.

**Table 1:** Univariate Cox proportional hazards regression analysis.

miRNA	LogFC	HR	z	*P*
**hsa-mir-424**	-1.47463701	1.731552246	3.781794216	0.000155702
**hsa-mir-3613**	-1.269297217	0.643105251	-2.819911816	0.004803685
hsa-mir-100	1.451158169	1.361225074	2.714421025	0.006639173
hsa-mir-139	-2.996528993	0.781057493	-2.595815983	0.009436659
**hsa-mir-4772**	-1.634160607	1.237295209	2.543043319	0.01098916
**hsa-mir-126**	-1.308472669	0.627370307	-2.527151064	0.011499203

Bold represents prognostic miRNAs.

**Table 2:** Multivariate Cox proportional hazards regression analysis.

miRNA	Coef	Exp (Coef)	SE (Coef)	z	*P*
**hsa-mir-424**	0.6006	1.8232	0.1646	3.648	0.000264
**hsa-mir-3613**	-0.3851	0.6804	0.1733	-2.223	0.026234
**hsa-mir-4772**	0.1819	1.1995	0.1047	1.737	0.082428
**hsa-mir-126**	-0.6601	0.5168	0.2027	-3.257	0.001126

### Prediction of the Target Genes of the Prognostic miRNAs

The online tools TargetScan and miRDB were used to predict the target genes of the four prognostic miRNAs, and the intersecting genes predicted by both databases were considered candidate target genes. A total of 6521 target genes were obtained (1081 target genes of hsa-mir-424, 692 target genes of hsa-mir-4772, 1243 target genes of hsa-mir-126 and 3505 target genes of hsa-mir-3613). After excluding 1384 duplicate target genes that were jointly regulated by multiple miRNAs, the total number of target genes of the four miRNAs was 5137. Then, we took the intersection of the 5137 target genes and the DEGs, and 118 common genes were identified (**Table 3**).

**Table 3:** Regulatory relationships between the prognostic miRNAs and common genes.

miRNA	Common gene				
**mir-424**	BTG2	PTPRR	EPB41L4B	PDCD4	KIF23
	KCNN4	SLC4A4	**COL12A1**	ESRRG	C2CD4B
	SLC7A2	AHNAK2	MTMR11	GLS2	ANLN
	PGM2L1	PDK4	TMC7	NRP2	BACE1
**mir-3613**	PTPRR	EPB41L4B	MET	LRRN1	**COL1A1**
	PDCD4	FNDC1	KIAA1324	EPHA4	LONRF2
	SDR16C5	OLR1	TRHDE	NR4A3	CD109
	LMO7	IAPP	**MMP14**	MCOLN3	MPP6
	GABRP	VCAN	ATRNL1	**COL11A1**	KCNJ16
	SLC4A4	AGR2	FOXQ1	DGKH	CCDC141
	SGIP1	ARNTL2	SV2B	UNC79	ITGB6
	SLC1A2	MATN3	GPRC5A	**COL3A1**	PRKAR2B
	INPP4B	NQO1	IFI44L	NR5A2	TNS4
	FLRT2	NRCAM	**COL6A3**	FBXO32	DPP10
	MTMR11	MBOAT2	SLC16A10	RTKN2	IGF2BP3
	PCDH7	SLC6A6	EDNRA	PROX1	**COL5A2**
	PDK4	IGFBP5	EFNA5	ADAM28	TMEM97
	SCG3	FAM129A	SCGN	NPR3	DCDC2
	ABHD17C	TMEM45B	ASPM	LIFR	**THBS2**
	PAIP2B	PLAC8	MMP9		
**mir-4772**	**ITGA2**	NR4A3	LMO7	SLC30A8	MMP6
	**COL12A1**	FOXQ1	SV2B	PAK3	F8
	PRKAR2B	FGD6	MBOAT2	RTKN2	ASAP2
	NPR3	FREM1			
**mir-126**	EPB41L4B	MLPH	TGFBI	DKK1	GJB2
	SCEL	REG3G	DCDC2	FAM129A	ETV1
	ANGPTL1	PKHD1	PGM2L1	PCDH7	TFPI2
	FABP4	FLRT2	ADAM9	ITGB6	SGIP1
	ESRRG	DGKH	BAIAP2L1	ESM1	ST6GALN
	**COL12A1**	KCNJ16	**COL11A1**	ADHFE1	AC1
	HOXB5	LONRF2	ACADL		

Bold represents key genes.

### Functional Enrichment Analysis of Common Genes

**Figure 3:**
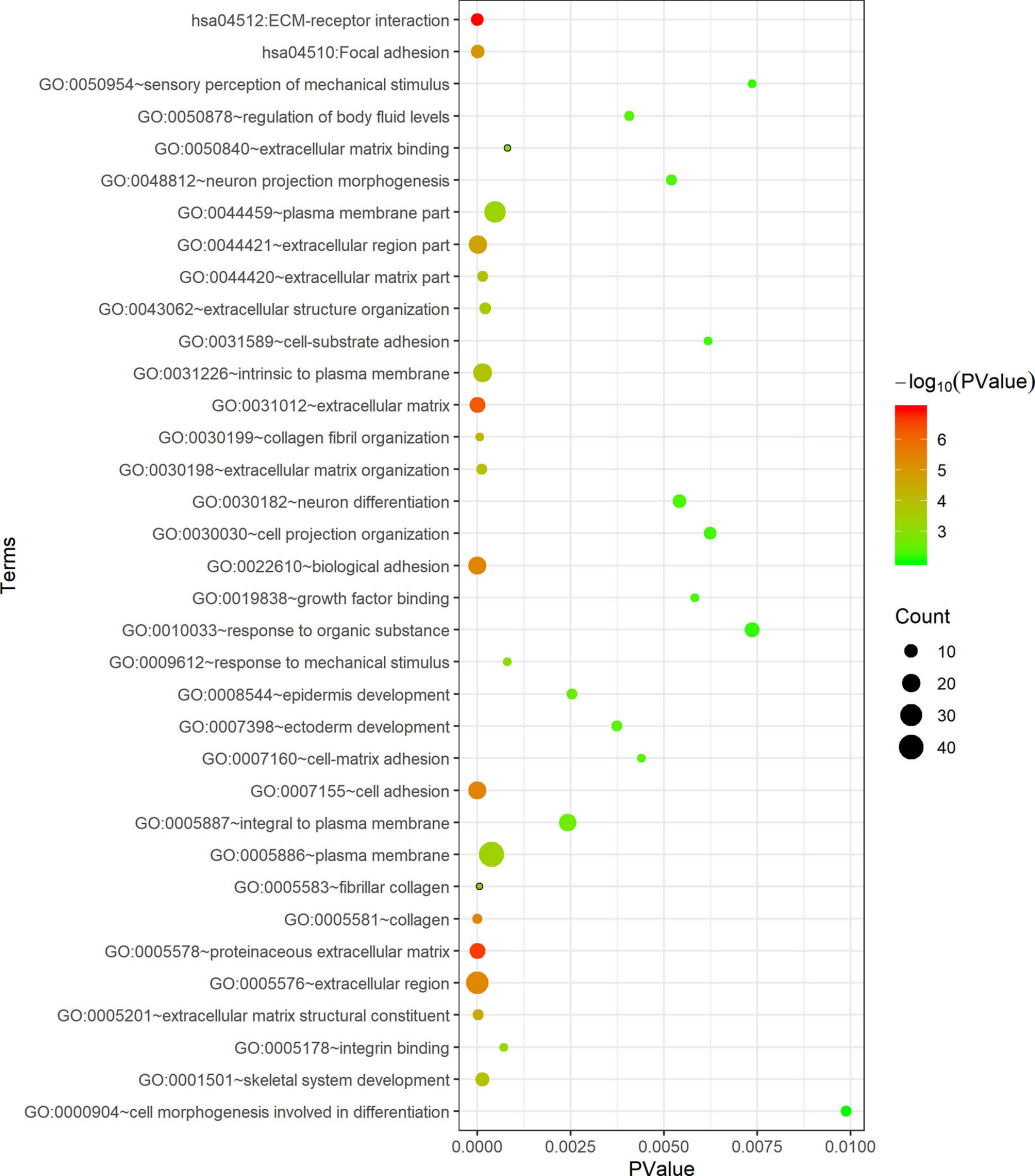
Functional enrichment analysis of 118 common genes. The x-axis represents the *P* value, and the y-axis represents the pathways and annotations. The bubble size increases with the number of enriched genes.

The DAVID database was applied for the KEGG pathway and GO functional annotation analyses of 118 common genes. Common genes were enriched in two KEGG pathways and 33 GO terms (*P* < 0.05) (**Figure 3**). The pathway with the smallest *P* value was ECM-receptor interaction (*P* = 1.04E-07), and the pathway with the largest count was focal adhesion (count = 10). In the BP category of GO, the common genes were mainly enriched in functional annotations such as cell adhesion, biological adhesion, skeleton system development, response to organic substance and sensory perception of mechanical stimulus. The annotation with the smallest *P* value in the BP category was cell adhesion (*P* = 3.72E-06), and the annotations with the largest count were cell adhesion (count = 19) and biological adhesion (count = 19). In the CC category, the common genes were mainly enriched in functional annotations such as proteinaceous extracellular matrix, extracellular matrix, extracellular region, intrinsic to plasma membrane and plasma membrane. The annotation with the smallest *P* value in the CC category was proteinaceous extracellular matrix (*P* = 2.30E-07), and the annotation with the largest count was plasma membrane (count = 42). In the MF category, the common genes were enriched in functional annotations such as extracellular matrix structural constituent, integrin binding and growth factor binding. The annotation with the smallest *P* value and the largest count in the MF category was extracellular matrix structural constituent (*P* = 2.65E-05, count = 7).

### PPI Network Construction and Key Gene Acquisition

**Figure 4:**
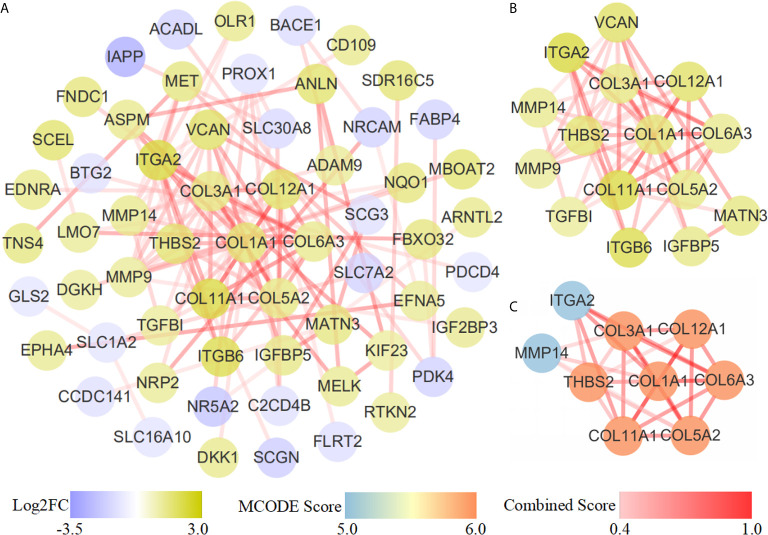
PPI network diagram. **(A)** PPI network of 118 common genes. **(B)** Results of the cytoHubba topological analysis. Different node colors represent the logFC value of the DEGs. **(C)** MCODE network module diagram. The shade and shallowness of the red lines represent the combined score between proteins.

The STRING database was applied to construct an interaction network of the 118 common genes (**Figure 4A**). The network contained 60 nodes and 107 edges. Using the MCC algorithm of the cytoHubba plug-in, the top 15 genes were identified (**Figure 4B**). The MCODE plug-in revealed one important functional module in the interaction network (MCODE score = 7.500, **Figure 4C**) that included nine key genes: MMP14, ITGA2, THBS2, COL1A1, COL3A1, COL11A1, COL6A3, COL12A1 and COL5A2. All nine key genes were upregulated in PDAC.

### Visualization of the miRNA-Gene-Pathway and Annotation Networks

**Figure 5:**
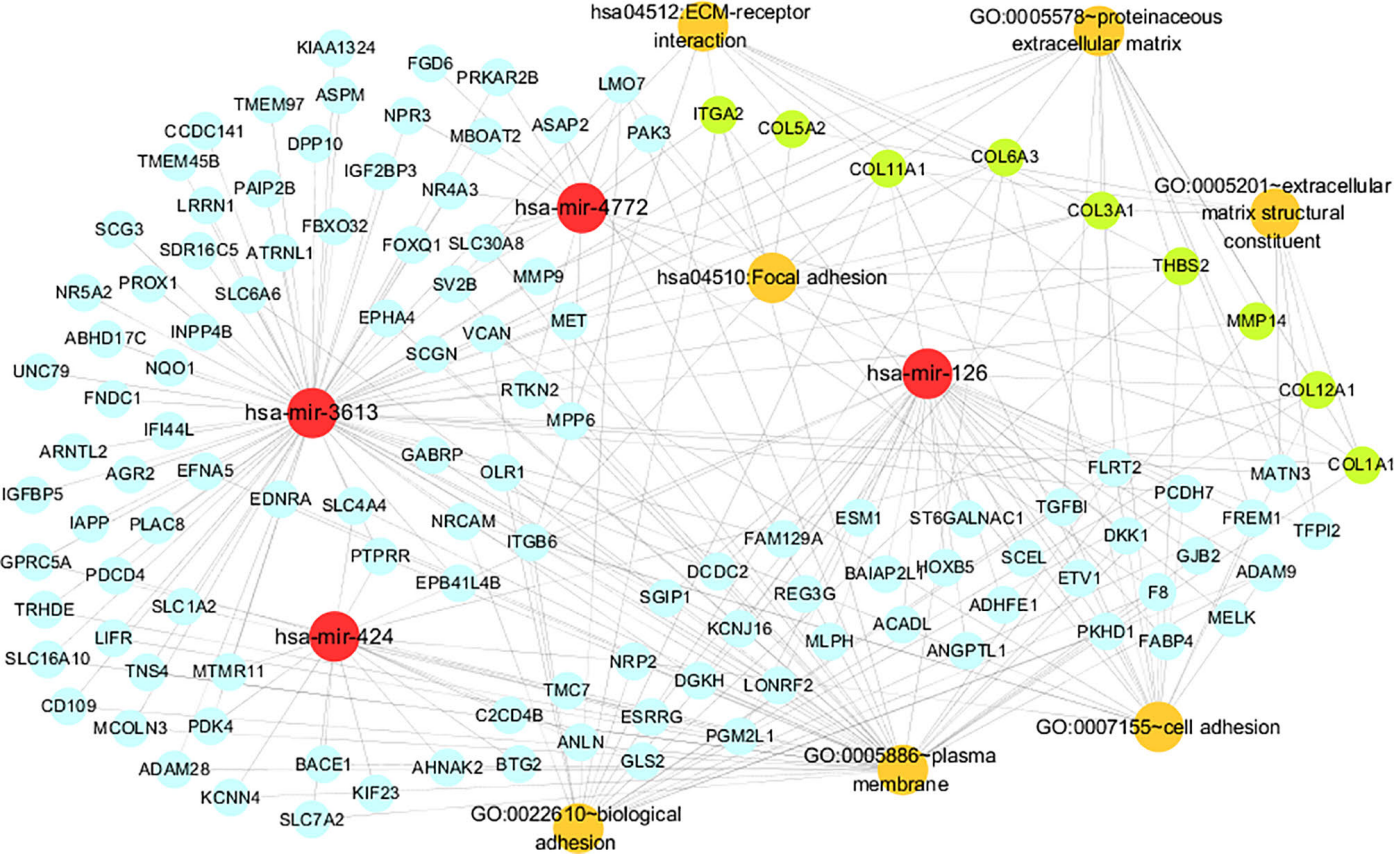
The miRNA-gene pathway and annotation networks represent the relationships among miRNAs, common genes, key genes and gene enrichment results. MiRNAs, common genes, key genes and enrichment terms are represented by red, blue, green and orange circles, respectively.

The miRNA-gene-pathway and annotation networks demonstrated the regulatory relationships between the miRNAs and key genes, as well as the enriched pathways and annotations of the key genes (**Figure 5**). Among them, hsa-mir-424 regulated COL12A1; hsa-mir-3613 regulated COL11A1, COL6A3, COL5A2, COL3A1, COL1A1, MMP14 and TSBH2; hsa-mir-4772 regulated COL12A1 and ITGA2; hsa-miR-126 regulated COL12A1 and COL11A1. KEGG pathway analysis indicated that COL6A3, COL3A1, ITGA2, COL1A1, COL5A2, THBS2, and COL11A1 were involved in the ECM-receptor interaction pathway; and COL6A3, COL3A1, ITGA2, COL1A1, COL5A2, THBS2, and COL11A1 were involved in the focal adhesion pathway. Regarding the GO annotation, COL3A1, ITGA2, COL6A3, COL12A1, THBS2, and COL11A1 were enriched in the biological adhesion and cell adhesion (GO-BP); COL3A1, COL12A1, COL1A1, COL5A2, and COL11A1 were enriched in the extracellular matrix structural constituent (GO-MF); and COL6A3, MMP14, and COL1A1 were enriched on the plasma membrane (GO-CC); and COL3A1, MMP14, COL5A2, COL6A3, COL12A1, COL1A1, and COL11A1 were enriched on the proteinaceous extracellular matrix (GO-CC).

### Establishment of Prognostic Model Based on the Key Genes

**Figure 6:**
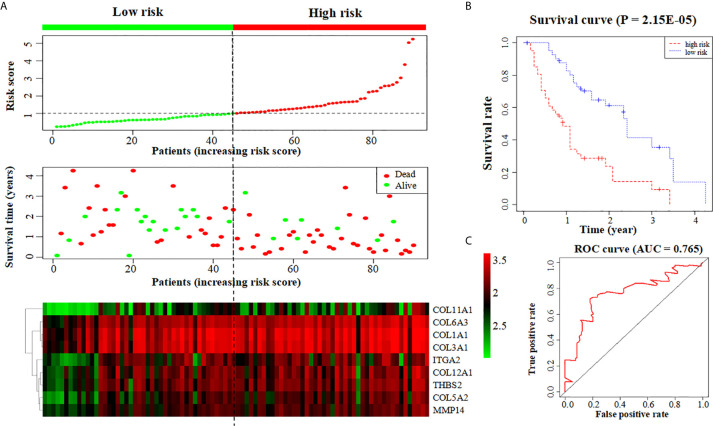
Construction of a prognostic model based on nine key genes. **(A)** The risk score curve, survival status and heatmap are shown from top to bottom. **(B)** Survival curve. **(C)** ROC curve of the prognostic model.

We further put the nine key genes into one prognostic model, and its prognostic risk score was calculated according to the following formula: Risk score = (-3.290e-01 × COL11A1) + (-1.358e+00 × COL12A1) + (3.811e+00 × COL1A1) + (-1.293e+01 × COL3A1) + (3.860e+00 × COL5A2) + (1.044e+01 × COL6A3) + (-2.232e-01 × ITGA2) + (4.880e+00 × MMP14) + (-4.655e+00 × THBS2). Then, the samples were divided into a high-risk group and a low-risk group based on the medium risk score. The risk score curve, survival status map and heatmap were plotted (**Figure 6A**). High risk score correlated with a poor prognosis. Survival analysis showed that the mortality rate increased as the risk score increased. Compared with high-risk group, low-risk group survival rate was notably higher (P = 2.15e-05; three-year survival rate, low-risk group: 35.50%, 95% CI = 20.21%-62.30%; high-risk group: 9.52%; 95% CI = 2.75%-32.9%) (**Figure 6B**). The AUC value of the ROC curve of the model was 0.765 (**Figure 6C**), which also possessed strong prognostic ability, indicating that these nine key gene we screened out were quiet reliable.

## Discussion

PDAC is a fatal digestive tumor that is difficult to diagnose and treat and is associated with a very poor prognosis (22); therefore, it is very important to identify novel molecular biomarkers or therapeutic targets. MiRNAs collectively act on protein-coding genes and are the main regulators of vital biological processes, such as cell proliferation, apoptosis, virus infection and carcinogenesis. Therefore, miRNAs have also become the focus of research in the field of tumor development.

To identify new credible prognostic miRNAs and important regulatory genes of PDAC, we screened 22 DEMs and 402 DEGs related to PDAC from the TCGA and GEO databases. Using Cox proportional hazards regression analysis and survival analysis, we obtained four miRNAs that are closely related to PDAC (hsa-mir-424, hsa-mir-4772, hsa-mir-126 and hsa-mir-3613) and incorporated them into a four-miRNA prognostic model with an AUC value of the survival ROC curve of 0.78. Then, 5147 target genes of these miRNAs were obtained from TargetScan and miRDB prediction, and 118 genes in the intersection of the target genes and DEGs were defined as common genes. Finally, the common genes were analyzed with STRING and Cytoscape plug-ins, and nine key genes (MMP14, ITGA2, THBS2, COL1A1, COL3A1, COL11A1, COL6A3, COL12A1 and COL5A2) related to PDAC were further acquired. We also constructed a prognostic model formed by these nine key genes, and the AUC value of its survival ROC curve was 0.765, indicating that these nine key genes screened out were quite reliable.

All four prognostic miRNAs (hsa-mir-424, hsa-mir-3613, hsa-mir-4772 and hsa-mir-126) are downregulated in PDAC according to our analysis. Among them, hsa-mir-126, hsa-mir-3613 and hsa-mir-424 have been experimentally verified to be underexpressed in PDAC. However, the downregulation of hsa-mir-4772 in PDAC has yet to be experimentally confirmed.

Previous studies have already proven that downregulated mir-126 and mir-3613 are involved in the development of PDAC, which agrees with our prediction. Hamada et al. (23) illustrated that in pancreatic cancer tissues, miR-126 is notably downregulated, while ADAM9 (a disintegrin and metalloprotease 9), the target gene of miR-126, is significantly upregulated. miR-126 upregulation induces ADAM9 suppression to further repress the metastasis and invasion ability of PDAC cells (23). Jiao et al. (24) also demonstrated inhibited miR-126 expression in PDAC tissue, and by increasing miR-126 expression, its target gene KRAS was repressed, thereby inhibiting the occurrence and development of PDAC. Jiang et al. (25) showed that low miR-3613 expression leads to a poor prognosis in PDAC patients, and their functional experiments demonstrated that miR-3613-5p inhibits proliferation and enhances the apoptosis of PDAC cells, indicating that hsa-miR-3613 acts as a tumor suppressor gene (25). All these results prove that the low expression of hsa-mir-126 and hsa-miR-3613 in pancreatic cancer tissue is related to the carcinogenesis of pancreatic cancer, but the specific mechanisms involved need to be further studied.

It has also been reported that mir-424 is abnormally expressed in PDAC; the results indicate that miR-424 suppresses PDAC development, which is inconsistent with our prediction. Wu et al. (26) found that miR-424-5p is upregulated in PDAC tissue compared to that in normal pancreatic tissue. Overexpressed miR-424-5p suppresses its target gene SOCS6 (suppressor of cytokine signaling 6), which further inhibits the expression of BCL-2 and MCL-1 in the ERK1/2 pathway, while downregulating overexpressed miR-424-5p inhibits the proliferation, migration and invasion of PDAC cells and promotes their apoptosis (26). These findings were inconsistent with our predictions in PDAC; however, it has been reported that miR-424 is downregulated in other cancers, in agreement with our calculations. For example, Fang et al. (27) showed that miR-424 is downregulated in colorectal cancer cells and patient biopsy tissues and that the upregulation of miR-424 can lead to proliferation inhibition and apoptosis induction in colorectal cancer cells. Wang et al. (28) showed that miR-424-5p expression is repressed in both tissues and cell lines of basal breast cancer and that low miR-424-5p expression is significantly associated with an advanced malignant status; however, the upregulation of miR-424-5p inhibits the proliferation and motility of basal breast cancer cells. Piao et al. (29) illustrated that miR-424-5p levels were significantly lower in liver cancer tissues than in normal liver tissues, while an increase in miR-424-5p levels can inhibit the expression of its target gene YAP1, leading to proliferation inhibition and apoptosis induction. Dong et al. (30) demonstrated that miR-424 is downregulated in endometrial cancer tissues compared to normal tissues and that the overexpression of miR-424 inhibits invasion and sphere formation in endometrial cancer cells. The above research shows that hsa-mir-424 can act as a tumor suppressor. The disagreement concerning the role of hsa-mir-424 in PDAC and other cancers in the literature aroused our interest. Thus, we carried out an experiment in which real-time quantitative PCR was applied to detect the expression level of mir-424 in the pancreatic cancer cell lines SW1990 and PANC-1 and the normal pancreatic epithelial cell line HPDE6C7. The experiments showed that hsa-mir-424 expression was significantly lower in PDAC cells than in normal cells (P<0.001), confirming our calculation (i.e., that hsa-mir-424 was indeed downregulated in PDAC cells; supplementary file, **Figure S1**). We cannot explain the difference between the results obtained by Wu et al. (26) and those obtained by us, but the expression of hsa-mir-424 in pancreatic cancer and its specific mechanism in the occurrence and development of pancreatic cancer are worthy of further investigation.

To date, no relevant experiment has proven that hsa-mir-4772 plays a role in PDAC; nevertheless, one bioinformatics study proposed that mir-4772 affects PDAC carcinogenesis, concordant with our results. Gupta et al. (31) illustrated the downregulation of miR-4772 in pancreatic cancer tissue through differential analysis and survival analysis of samples from the TCGA database and concluded that miR-4772 can be used as a prognostic marker to detect early pancreatic cancer. Thus, mir-4772 may suppress PDAC progression, bit its specific mechanism in PDAC is worthy of further exploration.

According to our prediction, the expression of nine key genes related to the prognosis of pancreatic cancer (MMP14, ITGA2, THBS2, COL1A1, COL3A1, COL11A1, COL6A3, COL12A1 and COL5A2) was upregulated. Among these proteins, the overexpression of MMP14, ITGA2, THBS2, COL1A1, COL3A1, COL11A1 and COL6A3 has been experimentally confirmed in PDAC, while that of COL12A1 and COL5A2 has been shown in other types of cancer. **Table 4** shows the expression of these nine key genes that have experimentally verified in PDAC and other cancers and whether they are consistent with our prediction results in PDAC.

**Table 4:** Features of key genes in previous studies.

Gene	MMP14	ITGA2	THBS2	COL1A1	COL3A1
Feature	▲	▲	▲	▲	▲
Gene	COL11A1	COL6A3	COL12A1	COL5A2	
Feature	▲	▲	△	△	

▲ These genes were experimentally determined to be upregulated in PDAC, and their expression was consistent with our prediction in PDAC. △ These genes were experimentally determined to be upregulated in other cancers, and their expression was consistent with our prediction in PDAC.

MMP14 (matrix metalloproteinase 14), also known as MT1-MMP, was the first member of the MMP family identified as a transmembrane protein (32). MMP14 is a collagenase that causes ECM degradation and leads to metastasis (32). Haage et al. (33)found that in pancreatic cancer, MMP14 promotes local ECM degradation and mediates cancer cell migration and invasion by activating MMP-2/9, while the inhibition of MMP14 suppresses cell migration (33). Dangi-Garimella et al. (34) demonstrated that the overexpression of MMP14 upregulates HMGA2, which increases the resistance of PDAC cells to the anticancer drug gemcitabine, subsequently leading to a poor prognosis. ITGA2 (integrin subunit alpha 2) is an important member of the integrin family (35). This gene encodes the transmembrane receptor alpha subunit of collagen and related proteins (35). ITGA2 affects cell proliferation, invasion, metastasis and angiogenesis in cancer. Ren et al. (36) demonstrated that ITGA2 is significantly upregulated in PDAC cells and tissues, and silencing overexpressed ITGA2 represses the progression of PDAC. They also demonstrated that ITGA2 upregulates the phosphorylation of STAT3, indicating that ITGA2 enhances proliferation and invasion by activating the STAT3 pathway (36). Gong et al. (37) illustrated that ITGA2 expression is elevated in PDAC cells, while miR-107 downregulates the expression of ITGA2 and suppresses the migration process by acting on the focal adhesion pathway. THBS2 (thrombospondin 2) is a member of the thrombospondin family (38). It is a disulfide-linked homotrimeric glycoprotein that mediates the interaction between cells and between cells and substrates (38). THBS2 is an effective inhibitor of tumor growth and angiogenesis. Kim et al. (39) showed that the THBS2 antigen is overexpressed in both cancer tissues and plasma of PDAC patients and might be related to the poor vascularization of PDAC. Le Large et al. (40)also revealed that THBS2 was significantly increased in pancreatic cancer tissues compared with normal tissues. In addition, both Kim (39) and Le Large (40) showed that the combined expression of THBS2 and CA19/9 in plasma can be used as a biomarker for PDAC patients. The above studies have shown that the overexpression of MMP14, ITGA2 and THBS2 promotes pancreatic cancer occurrence and development and indicate that they possess the ability to become therapeutic targets of PDAC.

COL1A1, COL3A1, COL11A1, COL6A3, COL12A1 and COL5A2 belong to the collagen family. The entire family contains 19 types of collagen and more than ten types of collagen-like proteins and is encoded by more than 30 different genes (41). Due to differences in its molecular structure, collagen can be divided into two categories: fibrogenic collagen and nonfibrogenic collagen (41). Types I, II, III, V, and IX collagen are fibrogenic (41). The extracellular matrix (ECM) is composed primarily of collagen, which is a macromolecular substance that supports the cell structure and regulates the physiological activities of the cell (42). ECM degradation is one of the most important steps that leads to cancer cell invasion and metastasis (43). Stably expressed collagen is necessary to maintain the normal functions of cells and tissues. However, the abnormal overexpression of collagen is associated with a variety of pathological processes, especially in malignant tumors.

COL1A1 is a member of the type I collagen family and encodes the pro-α 1 chains of type I collagen (44). Yang et al. (45) found that the miRNA sponge hsa-circRNA-0007334 inhibits hsa-mir-577 expression, leading to the overexpression of the mir-577 target gene COL1A1 and subsequently causing PDAC cell migration. They also demonstrated that high COL1A1 shortens the survival time of PDAC patients (45). COL3A1 is a type III collagenase whose lack causes perforation, tearing, fracture and even fragmentation of connective tissue-related structures in the body (46). Hall et al. (47) revealed that the overexpression of COL3A1 in patients with pancreatic cancer can be significantly downregulated after gemcitabine combined with EC359 treatment. COL11A1 is a specific type XI collagen cleavage fragment. This gene encodes one of the two α chains of type XI collagen (48). It comprises a major part of the ECM and affects the occurrence and development of a variety of cancers (49, 50). Kleinert et al. (51) reported that COL11A1 was significantly increased (by 5.52 times) in subjects with pancreatic cancer relative to those with normal chronic pancreatitis. Overexpression of the COL11A1 protein may be related to connective tissue proliferation events and can shorten the survival time of PDAC patients (51). García-Pravia et al. (52) also demonstrated that the COL11A1 gene was significantly overexpressed in subjects with pancreatic cancer relative to those with chronic pancreatitis, and they further revealed that the detection of proCOL11A1 by immunostaining can accurately distinguish PDAC from chronic pancreatitis. COL6A3, as a type VI collagen α chain, can interact with various components in the ECM (53). Its abnormal expression in various cancers suggests that COL6A3 affects cancer formation. Svoronos et al. (54) showed that in the PDAC stroma, COL6A3 is the major overexpressed gene, and overexpressed COL6A3 leads to a poor prognosis in PDAC. Arafat et al. (55) also showed that a high level of COL6A3 expression is found in the tissues of PDAC patients, especially those in later disease stages, and that patients in earlier stages present relatively low COL6A3 levels. The above experimental results indicate the oncogenic roles of COL1A1, COL3A1, COL11A1 and COL6A3 in PDAC formation and suggest that they may also possess potential as therapeutic targets of PDAC.

Although the upregulation of COL12A1 and COL5A2 has not been experimentally shown in pancreatic cancer, their abnormal overexpression has been experimentally shown in other cancer types. COL12A1 is encoded by a gene at chromosome position 6q12-q13 (56). COL12A1 connects fibers, and its mutation can cause muscle diseases (56). COL12A1 overexpression has been proven in several cancers, indicating its oncogenic role in human cancer. Jiang et al. (57) demonstrated that COL12A1 is upregulated in gastric cancer. The overexpression of COL12A1 is also associated with invasion, lymph node metastasis, distant metastasis and an advanced TNM stage of gastric cancer (57). Zhang et al. (58) first demonstrated COL12A1 overexpression in colorectal cancer cells using bioinformatics and then validated their results experimentally. The COL5A2 gene encodes a 46 kDa nuclear localization transcriptional inhibitor protein that has been reported to affect cancer progression (59). Fischer et al. (60) illustrated that in normal colon tissue, COL5A2 is not expressed, but in colon cancer tissues, COL5A2 is expressed. Chen et al. (61) showed that in osteosarcoma, COL5A2 expression can be repressed by the tumor suppressor gene NKX2-2. Thus, these two genes are upregulated in other cancers and have a great impact on the progression of those cancers, indicating that they may also affect the progression of PDAC in the same manner. However, the role and mechanism of COL12A1 and COL5A2 in PDAC deserve further study.

As shown in **Table 3**, there were 12 targeting relationships between the 4 prognostic miRNAs and 9 key genes. Specifically, COL12A1 is the target gene of hsa-mir-424; COL12A1 and COL11A1 are the target genes of hsa-mir-126; MMP14, TSBH2, COL3A1, COL1A1, COL11A1, COL6A3 and COL5A2 are the target genes of hsa-miR-3613; and ITGA2 and COL12A1 are the target genes of hsa-mir-4772. To date, none of the abovementioned targeting relationships have been confirmed experimentally. Therefore, the targeting relationships between these miRNAs and their target genes need to be confirmed in future studies.

In the KEGG pathway analysis of the 9 key genes, the ECM-receptor interaction pathway was enriched, and there are reports that this pathway can affect cell proliferation and differentiation, adhesion and metastasis (62). MMP14 and COL family genes are involved in tumor ECM regulation, which is very important for the study of cancer lymph node metastasis (32, 63). Therefore, it can be boldly speculated that MMP14 and these key COL family genes affect metastasis by acting on the ECM-receptor interaction pathway in pancreatic cancer. Regarding the GO functional annotations, cell adhesion has a profound impact on tumor proliferation and metastasis. Cell adhesion and connection maintain the integrity of the endothelial barrier, and malignant cancer cells metastasize through the blood or lymphatic vessels as soon as endothelial cells are impaired (64). MMP14 and ITGA2 are involved in cell adhesion according to previous studies. Munaut et al. (65) revealed that overexpressed MMP14 promotes the migration and invasion of glioblastoma cells by activating MMP-2 and upregulating VEGF. Ren et al. (36) also noted that ITGA2 reduces the adhesion of malignant tumor cells by acting on the STAT3 signaling pathway. Therefore, by affecting cell adhesion, MMP14 and ITGA2 may cause lymph node metastasis in PDAC cells. In summary, ECM regulation and cell adhesion will become the key to studying the mechanism of PDAC.

We also conducted bioinformatics analysis of mature miRNAs related to pancreatic cancer in the TCGA database in the same manner described above (**Figure S2**, **Table S1**). Analysis of the mature miRNAs revealed three miRNAs that can be used to predict the prognosis of pancreatic cancer patients, namely, hsa-miR-126-3p, hsa-miR-424-5p, and hsa-miR-3613-5p (**Table S2**). The mature prognostic model basically correlates with the precursor prognostic model, which also includes hsa-miR-4772 as an independent prognostic factor, while the mature model can distinguish between the 3p and 5p arms of miRNAs. The AUC value of the ROC curve was 0.784, which indicated that the prognostic ability of the mature miRNA prognostic model was slightly better than that of the precursor model (AUC = 0.78 (**Figure S3**). A mature miRNA contains 3p and 5p arms, limiting the prediction of the target genes of mature miRNAs and reducing the number of target genes. Therefore, by intersecting the mature miRNA target genes and DEGs of the GSE28735 dataset, only 28 common genes were obtained (**Table S3**). STRING analysis of the common genes showed that they are not very closely related, so a PPI network was not formed; therefore, the key genes were not obtained. That is, in this scenario, the precursor miRNAs provide more information than mature miRNAs. However, although the use of precursor miRNAs to construct a prognostic model may not be as reasonable as the use of mature miRNAs, our research shows that, consistent with previous experimental studies, prognostic models of precursor miRNAs and key genes are also reliable and accurate.

## Conclusions

In this study, by conducting a bioinformatics analysis on the miRNA and gene profiles of pancreatic cancer, we obtained a reliable four-miRNA (hsa-mir-424, hsa-mir-126, hsa-mir-3613 and hsa-mir-4772) prognostic model of PDAC. Further, nine key genes were identified: MMP14, ITGA2, THBS2, COL1A1, COL3A1, COL11A1, COL6A3, COL12A1 and COL5A2, which could also form an accurate prognostic model of PDAC. Among them, hsa-mir-4772, COL12A1 and COL5A2 were identified as novel PDAC biomarkers in PDAC that need to be experimentally proven. In addition, contrary to a previous study, mir-424 was confirmed to be downregulated in pancreatic cancer cells by qRT-PCR, agreeing with our prediction. These prognostic miRNAs and genes possess great potential as targets and biomarkers for PDAC treatment and prognosis. Our research can offer novel ideas for future diagnosis and treatment and may facilitate the development of new drugs.

## Data Availability Statement

The original contributions presented in the study are included in the article/**Supplementary Material**. Further inquiries can be directed to the corresponding authors.

## Author Contributions

ZH, GX, SC, and TY contributed to the design and conception of the study. SC, CG, TY, and YQ did information retrieval and analysis. SC, CG, and TY wrote the manuscript. SC, CG, TY, and YQ created tables and figures. ZH and GX guided manuscript writing and revision and provided financial support. All authors contributed to the article and approved the submitted version.

## Funding

This work was supported by the National Natural Science Foundation of China [31770774], the Provincial Major Project of Basic or Applied Research in Natural Science, Guangdong Provincial Education Department [2016KZDXM038], and the Higher Education Reform Project of Guangdong Province [2019268].

## Conflict of Interest

The authors declare that the research was conducted in the absence of any commercial or financial relationships that could be construed as a potential conflict of interest.

## Abbreviations

PDAC, pancreatic ductal adenocarcinoma; miRNA, microRNA; AMPKα1, AMP-activated protein kinase α1; TCGA, The Cancer Genome Atlas; GEO, Gene Expression Omnibus; DAVID, Database for Annotation, Visualization and Integrated Discovery; DEMs, differentially expressed miRNAs; DEGs, differentially expressed genes; PPI, protein-protein interaction; ADAM9, A disintegrin and metalloprotease 9; SOCS6, suppressor of cytokine signaling 6; MMP14, matrix metalloproteinase 14; ITGA2, integrin subunit alpha 2; THBS2, thrombospondin 2; COL1A1, collagen alpha-1(I) chain; COL3A1, collagen alpha-1(III) chain; COL11A1, collagen alpha-1(XI) chain, COL6A3, collagen alpha-3(VI) chain; COL12A1, collagen alpha-1(XII) chain; COL5A2, collagen alpha-2(V) chain; ECM, extracellular matrix.
