# Supplementary material for: Bioinformatics Analysis of a Prognostic miRNA Signature and Potential Key Genes in Pancreatic Cancer
Source: Front Oncol. 2021 May 20;11:641289. doi: 10.3389/fonc.2021.641289 (PMC8174116; doi:10.3389/fonc.2021.641289)
Supplement: Supplementary file 1 [file DataSheet_1.doc]

**Bioinformatics analysis of a prognostic miRNA signature and potential key genes in pancreatic cancer**

**Shuoling Chen^#^, Chang Gao^#^, Tianyang Yu^#^, Yueyang Qu, Gary Guishan Xiao*, Zunnan Huang***

1. **qRT-PCR of hsa-mir-424**

**
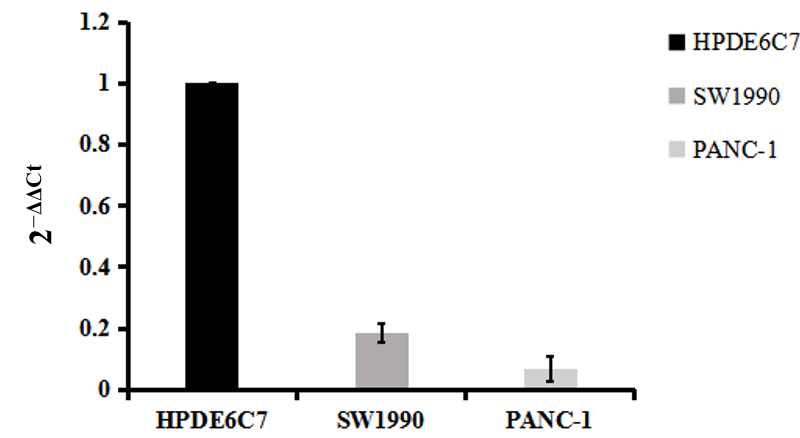
**

**Figure S1.** Expression levels of hsa-mir-424 in normal pancreatic cells (HPDE6C7) and pancreatic cancer cell lines (SW1990 and PANC-1) validated using qRT-PCR.

**2. Differential expression analysis**


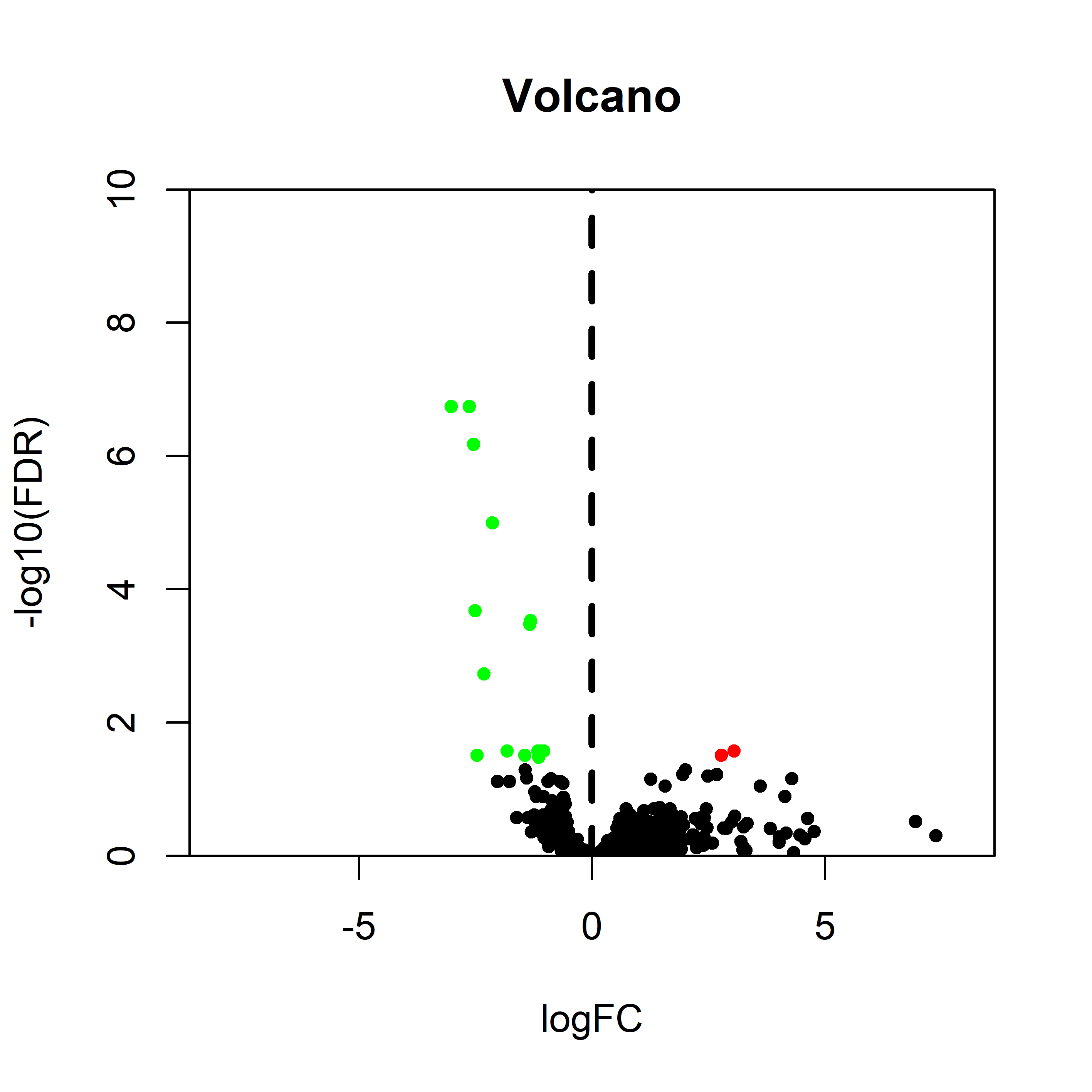
18 DEMs were identified from 183 PDAC samples of TCGA, 2 were up-regulated and 16 were down-regulated (Figure S2).

**Figure S2.** Volcano diagrams of DEMs (A). Green red circles represent downregulated and upregulated miRNA or genes, respectively.

**3. Construction of the Cox proportional hazards regression model**

Six miRNAs associated with survival of PDAC patient were identified (*P* < 0.05) (Table S1). Three downregulated prognostic miRNAs were further selected (hsa-miR-424-5p, hsa-miR-126-3p and hsa-miR-3613-5p) (Table S2). Their *P* values were less than 0.05, indicating that they were independent prognostic factors. The prognostic miRNA risk score was calculated according to the following formula: risk score = (0.617756388 × hsa-miR-424-5p) + (-0.543115715 × hsa-miR-126-3p) + (-0.467523356 × hsa-miR-3613-5p) . Then, the samples were divided into a high-risk groups and a low-risk group based on the medium risk score. The risk score of the former group was higher than that of the latter group (Figure S3A, top). A high risk score correlated with a poor prognosis. Survival analysis showed that the mortality rate increased as the risk score increased (Figure S3A, middle). The heatmap showed that as the risk score increased, the expression levels of hsa-miR-424-5p and hsa-miR-126-5p increased, indicating that they were high-risk miRNAs; the expression of hsa-miR-3613-5p decreased as the risk score increased, indicating that it was a low-risk miRNA (Figure S3A, bottom). Compared with high-risk group, low-risk group survival rate was notably higher (P = 2.87e-04; three-year survival rate, low-risk group: 53.6%, 95% CI = 42.10%-68.10%; High-risk group: 18.96%; 95% CI = 10.88%-33.00%) (Figure S3B). The AUC value of the ROC curve of the model is 0.784 (Figure S3C), which was greater than 0.7, indicating that the model was highly reliable.

**Table S1. Univariate Cox proportional hazards regression analysis**

| **miRNA** | **LogFC** | **HR** | **z** | ***P*** |
| --- | --- | --- | --- | --- |
| **hsa-miR-424-5p** | -1.3338026 | 1.804558764 | 3.771901083 | 0.000162008 |
| **hsa-miR-3613-5p** | -1.145937386 | 0.640651241 | -3.377840938 | 0.000730573 |
| hsa-miR-424-3p | -1.164039576 | 1.496568338 | 3.025484633 | 0.00248235 |
| **hsa-miR-126-3p** | -1.035741286 | 0.581590454 | -2.8303152 | 0.004650217 |
| hsa-miR-139-5p | -2.63155533 | 0.78073347 | -2.617709424 | 0.008852215 |
| hsa-miR-126-5p | -1.314991235 | 0.663485173 | -2.231526205 | 0.025646294 |

Note: Bold represents prognostic miRNAs.

**Table S2.** Multivariate Cox proportional hazards regression analysis

| **miRNA** | **coef** | **exp(coef)** | **se(coef)** | **z** | ***P*** |
| --- | --- | --- | --- | --- | --- |
| **hsa-miR-424-5p** | 0.617756388 | 1.854762004 | 0.16292528 | 3.791654614 | 0.000149647 |
| **hsa-miR-3613-5p** | -0.467523356 | 0.626552095 | 0.139840638 | -3.343258172 | 0.000828008 |
| **hsa-miR-126-3p** | -0.543115715 | 0.580935401 | 0.193812418 | -2.802275106 | 0.005074358 |

**
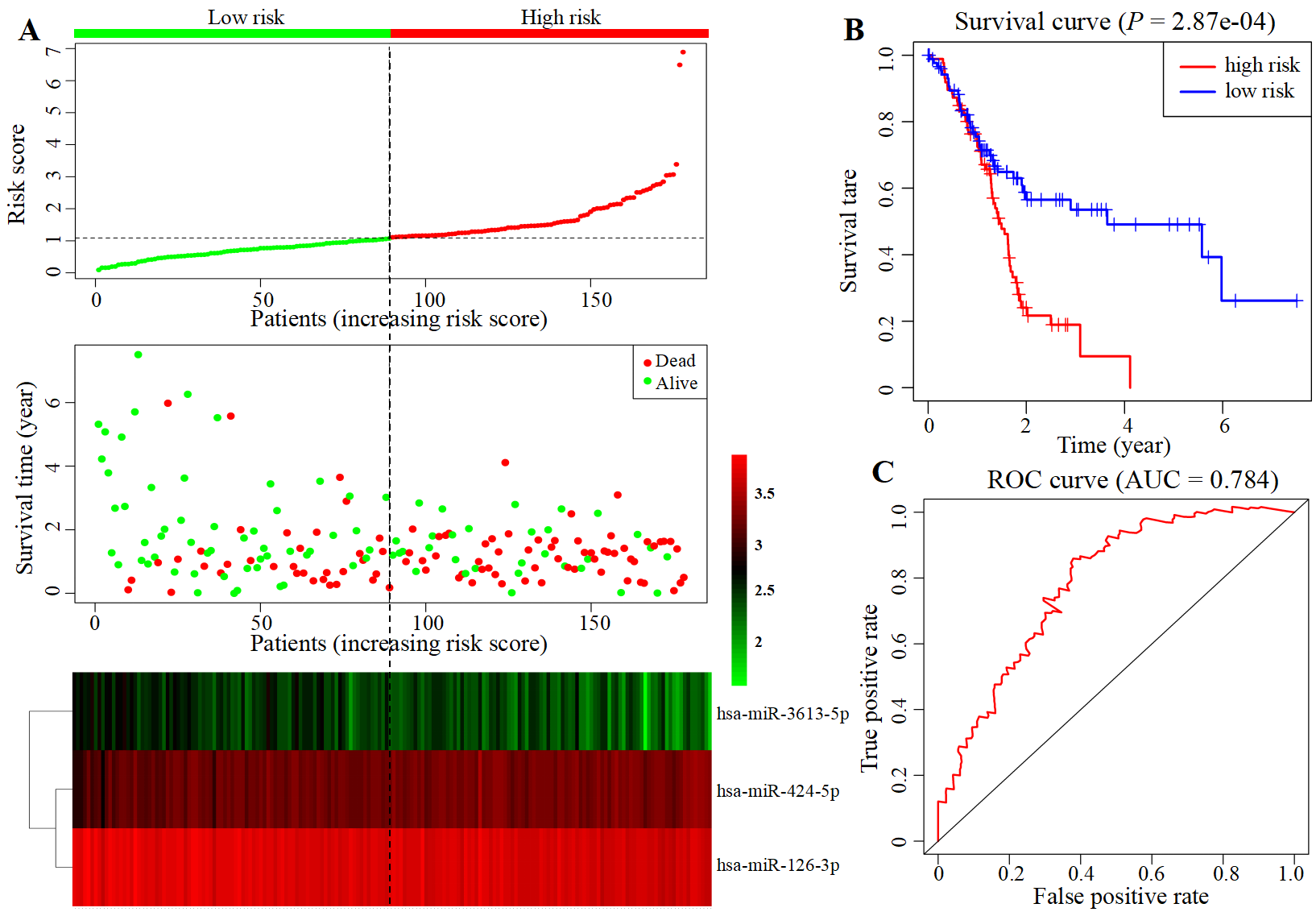
**

**Figure S3.** (A) The risk score curve, survival status and heatmap are shown from top to bottom. (B) Survival curve. (C). ROC curve of the prognostic model.

**3. Prediction of the target genes of the prognostic miRNAs**

The online tools TargetScan and miRDB were used to predict the target genes of the four prognostic miRNAs, and the intersecting genes predicted by the both databases were considered candidate target genes. A total of 1158 target genes were obtained (891 target genes of hsa-miR-424-5p, 13 target genes of hsa-miR-126-3p and 254 target genes of hsa-miR-3613-3p). After excluding 30 duplicate target genes that were jointly regulated by multiple miRNAs, the total number of target genes of the three miRNAs was 1128. Then, we took the intersection of 1128 target genes and the DEGs, and 28 common genes were identified (Table S3).

**Table S3.** The regulatory relationship between prognostic mature miRNAs and common genes

| **miRNA** | **Common gene** | | | |
| --- | --- | --- | --- | --- |
| miR-126-3p | ADAM9 |  |  |  |
| miR-424-5p | BTG2 | PTPRR | EPB41L4B | PDCD4 |
|  | ANLN | NRP2 | ESRRG | PGM2L1 |
|  | PDK4 | KIF23 | BACE1 | SLC7A2 |
|  | AHNAK2 | SLC4A4 | KCNN4 | MTMR11 |
|  | TMC7 | GLS2 | COL12A1 |  |
| miR-3613-5p | KIAA1324 | COL5A2 | AGR2 | MCOLN3 |
|  | NPR3 | ABHD17C | ASPM | FLRT2 |
